# Unveiling breast cancer metastasis through an advanced X-ray imaging approach

**DOI:** 10.1038/s41598-024-51945-4

**Published:** 2024-01-16

**Authors:** Andre L. C. Conceição, Volkmar Müller, Eike-Christian Burandt, Malte Mohme, Leonard C. Nielsen, Marianne Liebi, Sylvio Haas

**Affiliations:** 1 Deutsches Elektronen-Synchrotron DESY, Notkestr. 85, 22607 Hamburg, Germany; 2 Department of Gynecology, University Medical Center Hamburg-Eppendorf, Martinistraße 52, 20246 Hamburg, Germany; 3 Institute of Pathology, University Medical Center Hamburg-Eppendorf, Martinistraße 52, 20246 Hamburg, Germany; 4 Department of Neurosurgery, University Medical Center Hamburg-Eppendorf, Martinistraße 52, 20246 Hamburg, Germany; 5 Department of Physics, Chalmers University of Technology, 41296 Gothenburg, Sweden; 6 Photon Science Division, Paul Scherrer Institute, 5232 Villigen PSI, Switzerland; 7 Institute of Materials, Ecole Polytechnique Fédérale de Lausanne (EPFL), 1015 Lausanne, Switzerland

**Keywords:** Cancer imaging, Diagnostic markers, Imaging techniques

## Abstract

Breast cancer is a significant global health burden, causing a substantial number of deaths. Systemic metastatic tumour cell dissemination is a major cause of poor outcomes. Understanding the mechanisms underlying metastasis is crucial for effective interventions. Changes in the extracellular matrix play a pivotal role in breast cancer metastasis. In this work, we present an advanced multimodal X-ray computed tomography, by combining Small-angle X-ray Scattering Tensor Tomography (SAXS-TT) and X-ray Fluorescence Computed Tomography (XRF-CT). This approach likely brings out valuable information about the breast cancer metastasis cascade. Initial results from its application on a breast cancer specimen reveal the collective influence of key molecules in the metastatic mechanism, identifying a strong correlation between zinc accumulation (associated with matrix metalloproteinases MMPs) and highly oriented collagen. MMPs trigger collagen alignment, facilitating breast cancer cell intravasation, while iron accumulation, linked to angiogenesis and vascular endothelial growth factor VEGF, supports cell proliferation and metastasis. Therefore, these findings highlight the potential of the advanced multimodal X-ray computed tomography approach and pave the way for in-depth investigation of breast cancer metastasis, which may guide the development of novel therapeutic approaches and enable personalised treatment strategies, ultimately improving patient outcomes in breast cancer management.

## Introduction

Breast cancer remains a significant worldwide sorrow. It has a high incidence rate and a significant number of deaths reported globally. It has overtaken lung cancer as the most commonly diagnosed cancer, with an estimated 2.3 million new cases worldwide each year, and it is a leading cause of cancer-related mortality^1^. Among the various factors contributing to poor outcomes in breast cancer, metastasis remains a major cause of death^2,3^.

Metastasis formation is a complex cascade process involving the spread of cancer cells from primary tumours to distant organs^4,5^. Cancer cells' ability to invade, migrate and colonise secondary sites significantly affects patient prognosis. An understanding of the complex mechanisms underlying metastasis is critical to the development of effective interventions against and also the potential prevention of advanced breast cancer^6^.

Accumulating evidence suggests that alterations in the extracellular matrix (ECM)^7–9^, particularly alterations in collagen fibril remodelling, play a central role in breast cancer metastasis^10–12^. Collagen, a major structural component of the ECM, provides mechanical support and influences cellular behaviour in tissues. Perturbations in collagen fibril arrangement and composition have been associated with tumour progression, invasion and metastasis, making them attractive targets for therapeutic intervention^11,13–17^.

The identification of tumour-associated collagen signatures has emerged as a major research focus in the understanding of breast cancer metastasis^18–20^. These signatures include distinct patterns of collagen organisation characterised by altered fibre orientation, increased density and enhanced cross-linking within the tumour microenvironment. They provide insight into the dynamic interplay between cancer cells and the ECM that influences tumour behaviour and progression. While many insights into tumour-associated collagen signatures have been gained from cell culture experiments and animal models, there is a growing need to investigate and understand these phenomena in real tissue samples from breast cancer patients^21^. Exploring the native tissue environment in a three-dimensional way is critical to capture the complexity and heterogeneity associated with human breast cancer metastasis.

In addition to collagen remodelling, overexpression of matrix metalloproteinases (MMPs)^22–25^ and vascular endothelial growth factor (VEGF)^26–28^ have been implicated in breast cancer metastasis. MMPs facilitate ECM degradation, including collagen degradation, thereby promoting tumour cell invasion and migration. VEGF, on the other hand, plays a critical role in angiogenesis, enabling the formation of new blood vessels to support tumour growth and metastasis.

Although much has been learned about breast cancer initiation, progression and metastasis, experimental models are often limited to 2D cell culture or animal models. They do not necessarily reproduce the real physiological pattern of the human tumour microenvironment^29^. Recent advances in X-ray imaging techniques offer promising solutions for investigating tumour-associated collagen signatures and metal accumulation in breast cancer spatially resolved in 3D. Techniques based on X-ray scattering and emission, such as small-angle X-ray scattering tensor tomography (SAXS-TT)^30–33^ and X-ray fluorescence computed tomography (XRF-CT)^34,35^, allow the mapping of collagen orientation and the extraction of nanostructural parameters, as well as the metal distribution within tissues, respectively.

Small-angle X-ray scattering (SAXS) has been employed to elucidate the structural characteristics and organization of collagen in breast cancer^36,37^. Through the integration of computed tomography (SAXS-CT), a comprehensive three-dimensional mapping of collagen distribution in both benign and malignant human breast tumours has been achieved^38^. Advancing into the third dimension within reciprocal space, SAXS-TT facilitates the three-dimensional visualization of anisotropic nanostructures in a material, offering a non-destructive technique that provides six-dimensional mapping^31^. Therefore, this technique measures the directional dependence of scattered X-rays at small angles, providing detailed insights into the orientation and arrangement of nanoscale features within a given sample. Specifically, this study focuses on elucidating the rearrangement of collagen fibril bundles in breast tumour tissue. In addition, XRF-CT allows the visualisation and quantification of metal elements, such as zinc and iron, which have been implicated in breast cancer progression and metastasis^39^. These emerging imaging modalities have immense potential to provide a deeper understanding of the tumour microenvironment and its influence on metastatic processes.

In this study, we investigated changes in the 3D architecture of a solid breast cancer tumour to prove the potential of the advanced multimodal X-ray imaging approach to address the complex mechanism of breast cancer metastasis. By combining SAXS-TT and XRF-CT, we map simultaneously the remodelling of collagen fibrils as well as the accumulation of iron and zinc within the tumour microenvironment. Correlating these findings, we expect to gain valuable insights into the interplay between collagen remodelling and metal accumulation in the breast cancer metastasis cascade.

## Results and discussion

### Collagen fibre orientation

**Figure 1:**
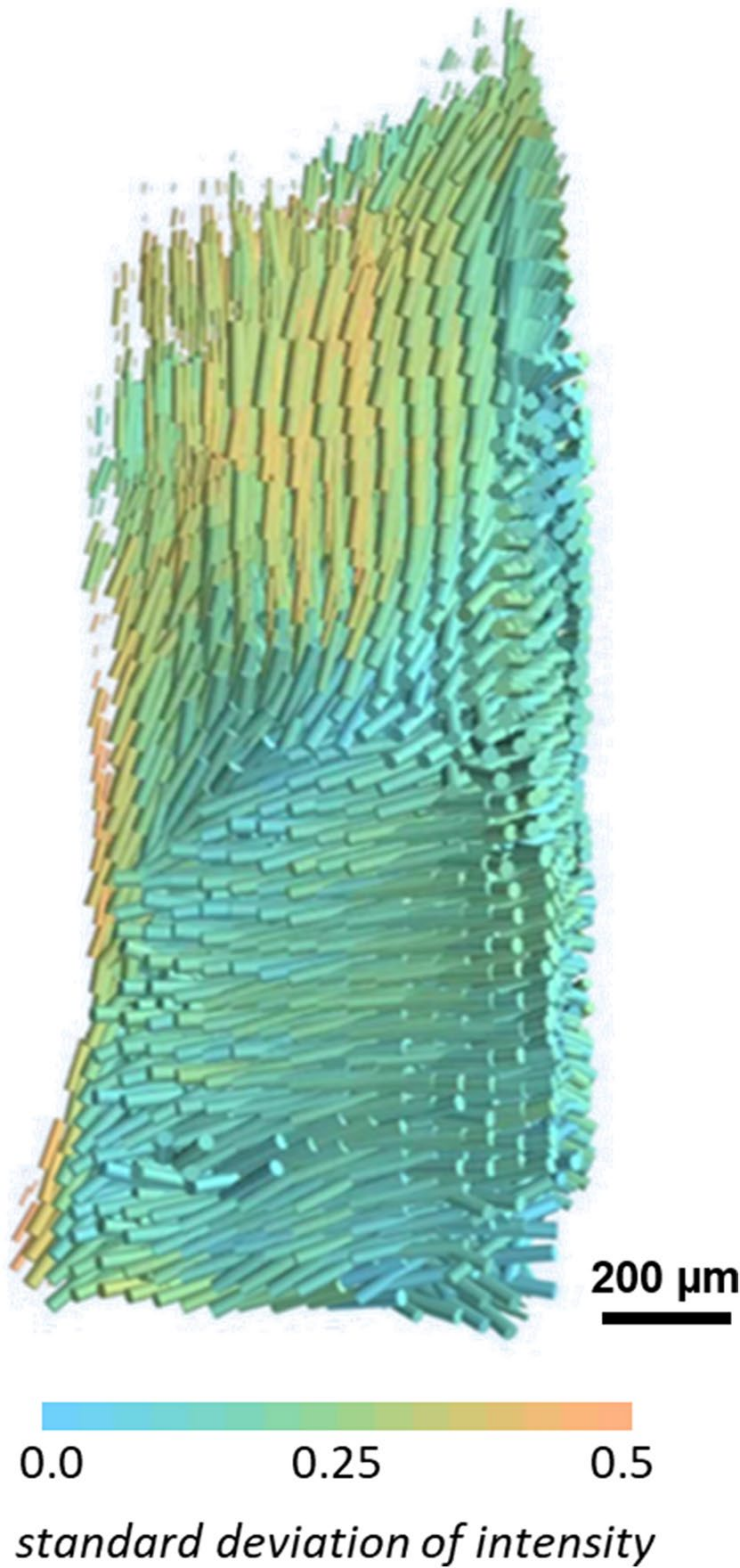
SAXS-TT Reconstructed orientation map of the collagen fibrils. 3D representation of the spatially resolved direction of the collagen anisotropy based on the SAXS evaluation at $q$ = 0.48 nm^−1^. The direction of the cylinders shows the direction of collagen orientation and the color scale the degree of orientation. Lower standard deviation means a weaker degree of orientation while higher standard deviation reflects stronger alignment of the collagen fibers in the voxel. Sample dimension: ~ 1.2 mm diameter and ~ 5.0 mm height.

One of the most prominent hallmarks of breast cancer is collagen, which increases tumour stiffness, regulates tumour immunity and promotes metastasis^40^. In the tumour microenvironment (TME), the content and architecture of collagen fibrils around the tumour are strongly altered by mutated tumour suppressor genes, such as p53, to further coordinate proliferation and invasion^21^. Cancer cells reverse-engineer collagen fibrils to form a reinforcing cell-collagen loop that gradually promotes cancer progression. Among these modifications, collagen I (COL1) present in the extracellular matrix (ECM) is of particular interest. In addition to a local increase in the density of this type of collagen around the tumour, the orientation of COL1 is essential in guiding tumour expansion and metastasis^17^. Because of this very important role, the prognostic factor associated with collagen orientation is called tumour-associated collagen signatures (TACS1-3)^41^. TACS1-3 predicts the behaviour of cancer cells according to collagen orientation. Therefore, the volumetric map of collagen orientation is essential for a better understanding of the tumour development process. The three-dimensional orientation map of collagen fibrils exhibited in Fig. 1 is based on their 5th-order reflection, identified as the more representative^36,37^. The colour bar is based on the degree of anisotropy in each reconstructed reciprocal space map, defined as the standard deviation of the intensity divided by the mean. In other words, orange means highly oriented collagen and cyan represents the disordered collagens.

**Figure 2:**
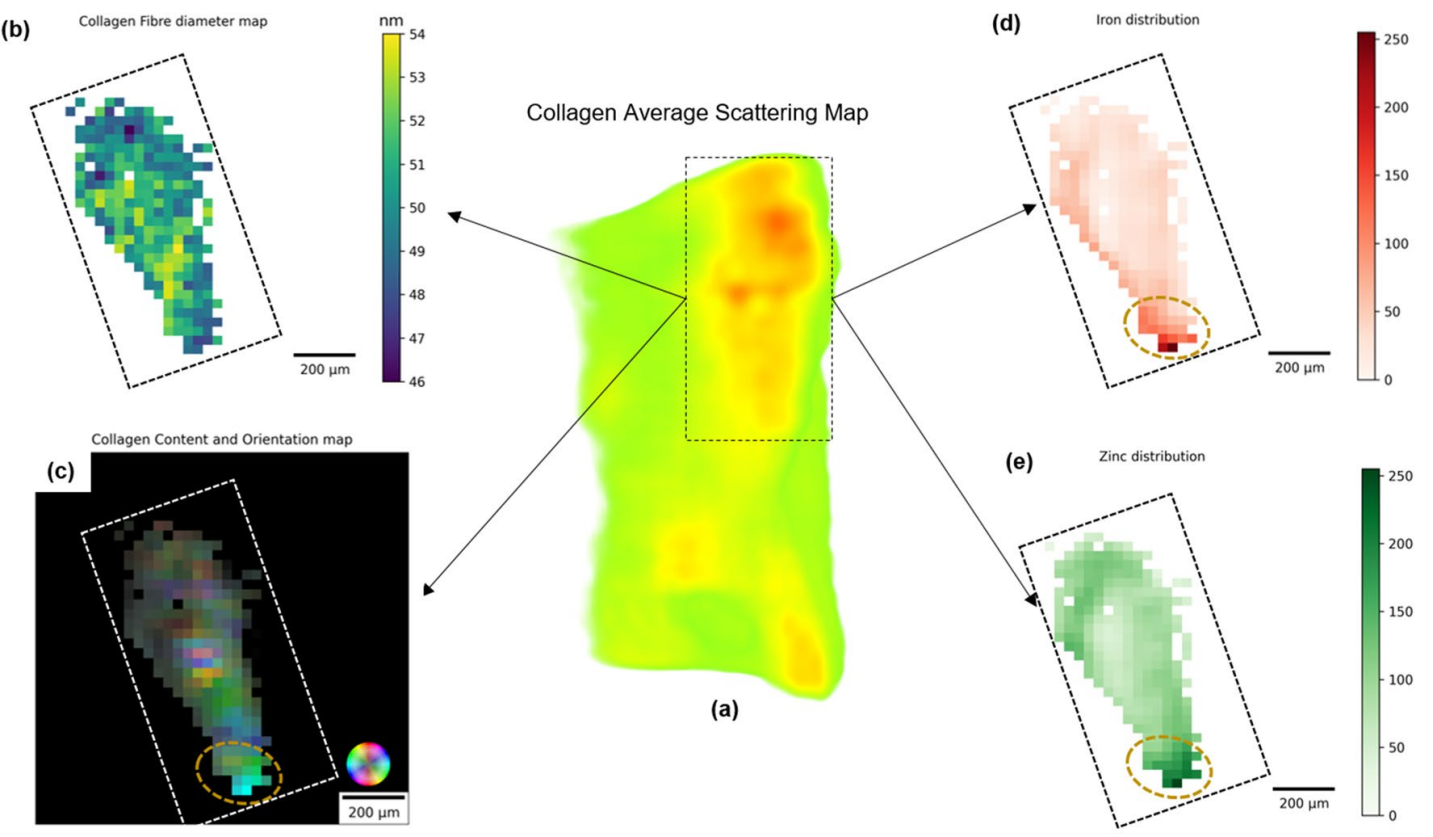
Multimodal X-ray tomography. (**a**) Three-dimensional map of the average scattering of the collagen fibrils, reconstructed at the $q$ = 0.48 nm^−1^. The dashed rectangle indicates the higher scattering intensity region. The green area inside the dashed rectangle was masked out for further maps due to none or very low collagen content; (**b**) the distribution of the calculated collagen fibril diameter within the region of interest (ROI); (**c**) the orientation map retrieved from the SAXSTT reconstruction in Fig. 1. The orientation is colour-coded according to the colour wheel inset. Highly oriented collagen fibrils show up with bright colours, whereas dense fibrils without preferential orientation are darker; (**d**) and (**e**) normalized distribution of Fe and Zn, respectively. The brown ellipse dashed area in (**c**), (**d**) and (**e**) highlights a potential metastatic site.

Figure 1 clearly shows a higher degree of orientation in the upper part of the sample (orange region) as opposed to the disordered collagen in the lower region. The localised aligned collagen may reflect a metastatic focus (TACS-3), regardless of whether the collagen fibrils are aligned parallel or perpendicular to the tumour. However, for a more in-depth evaluation, the reverse analysis approach^38^ was applied to this region. The reverse analysis approach enables performing further analysis using SAXS profiles retrieved from reconstructed voxels. From this analysis, quantitative information on nanostructure parameters such as the diameter of the collagen fibrils can be obtained. In addition, the corresponding zinc and iron accumulation maps were determined for this region. The reconstructed maps from multimodal X-ray fluorescence and small-angle scattering imaging are shown in Fig. 2 for the region of aligned collagen.

In Fig. 2a, the average collagen scattering map in 3D is enhanced in the upper right, a region of higher collagen scattering intensity reflecting an increase in the density and degree of orientation of collagen fibrils, reconstructed using its 5th-order reflection peak, conform orientation map in Fig. 1. The region of interest (ROI), the rectangular dashed area in Fig. 2a, was further investigated by applying the reverse analysis approach^38^ within this area to determine and map the collagen fibril diameter. The collagen fibrils are modelled as cylinders with axially periodic step-function electron density, packed as hexagonal bundles, as described by Suhonen et al.^42^. By fitting the model to the SAXS profiles, nanostructure features of the collagen in the breast tumour can be determined, such as axial periodicity and fibril diameter. The fibril diameter map was determined and presented in Fig. 2b. It shows the significant changes over the region and could also be used as a prognostic factor in breast cancer^43^. The calculated fibril diameter ranges from 46 to 54 nm. An increase in collagen fibril diameter is observed from the top to the bottom, surrounding the potential metastatic site, dashed, in Fig. 2c–e, as suggested by Sapudom et al.^44^. It has been shown that stromal expression of lysyl oxidase-like 2 (LOXL2), an important enzyme for collagen cross-linking, can enhance collagen cross-linking, resulting in higher collagen density, and is likely to increase the length and diameter of collagen fibres further from the tumour^45^. In addition, an inverse relationship was observed between collagen fibril diameter and 5-year overall patient survival.

The corresponding collagen orientation map retrieved from SAXS-TT (Fig. 1) for the dashed rectangular ROI in Fig. 2a is shown in Fig. 2c. Orientation is colour-coded according to the colour wheel inset, where brighter regions indicate a higher degree of orientation. A brighter area at the bottom of the sample orientation map is highlighted by a brown dashed ellipse. In this area, the majority of collagen fibrils are oriented perpendicular to the side plane, as shown by the cylinder direction for the corresponding region in Fig. 1. In addition to the tumour behaviour predicted by TACS1-3, aligned collagen fibrils around vascular regions and cancer cells also restrict the migration of T cells and limit their entry into the tumour mass^46^.

Figure 2d and e show the reconstructed maps of the distribution of iron and zinc, respectively, obtained from X-ray fluorescence computed tomography. The intensities are normalised (0–255) in such a way that higher values represent a higher accumulation of the elements. In the ellipse dashed area, in the lower part of the ROI, a higher concentration of Fe and Zn is identified. Iron accumulation has been reported in both early and late stages of tumour development^47–49^. The role of iron in cancer progression is closely linked to its interference with the immune response and angiogenesis. To control iron availability, macrophages and T-cells can modify their phenotype, similar to mechanisms observed in defence against pathogens, to release iron into the tumour microenvironment and promote tumour growth^50^. Tumour angiogenesis is characterised by the secretion of multiple pro-angiogenic factors to trigger the angiogenic switch, resulting in the development of a structurally and functionally abnormal vasculature. In this scenario, iron accumulation is directly related to the overexpression of vascular endothelial growth factor (VEGF), which promotes angiogenesis and consequently induces cell proliferation and metastasis^51^.

Increased zinc levels in tumours are associated with matrix metalloproteinases (MMPs) and the zinc-regulated transporter (ZRT), iron-regulated transporter (IRT)-like proteins (ZIPs). MMPs are a family of zinc-dependent endopeptidases involved in the degradation of the extracellular matrix (ECM)^23^. MMPs are upregulated in almost all human cancers and can promote cancer progression by increasing cancer cell growth, migration, invasion, metastasis and angiogenesis. In breast cancer in particular, MMP-8 preferentially degrades type I collagen, whereas MMP-2 and MMP-9 are known to degrade type IV collagen, which is particularly abundant in basement membranes^52^. Although MMPs are the most commonly studied zinc-dependent molecules in cancer, recent studies have shown that the sophisticated regulation of specific ZIPs plays an important role in the acquisition of a malignant phenotype in response to specific types of stimulation. An association between ZIP10 and metastasis in human breast cancer cells has been reported by Kagara et al^53^.

Figure 2 provides a comprehensive picture of the major breast cancer hallmarks in a real solid breast cancer tumour. Metastasis is a cascade process and involves the role of several essential molecules, from ECM remodelling, signalling pathways, migration of cancer cells from the primary organ to a secondary organ and finally the formation of distant metastatic tumour colonies. The influence of each of these key molecules in this complex mechanism is crucial. To provide insight into this complex mechanism, Table 1 quantifies the pairwise cross-correlation between each of the parameters examined in this study.

**Table 1:** Dynamic cross-correlation.

	Collagen orientation	Collagen diameter	Fe accumulation	Zn accumulation
Collagen orientation	1.00	0.81	0.77	0.88
Collagen diameter	0.81	1.00	0.70	0.84
Fe accumulation	0.77	0.70	1.00	0.89
Zn accumulation	0.88	0.84	0.89	1.00

Calculated pair-wise cross-correlation between collagen orientation and diameter, iron and zinc accumulation.

The cross-correlation between the features extracted from the reconstructed multimodal X-ray images (Table 1) is all positive and consistent with the reported findings observed in the culture cell or animal models. While Fe accumulation is weaker correlated with larger collagen fibril diameter, higher zinc concentration is strongly correlated with aligned collagen. As the increase in iron in the tumour region is mainly related to angiogenesis, interference with the immune response and, to some extent, zinc transport, it is expected that a higher correlation of iron with zinc accumulation would be observed rather than with collagen reorientation and diameter. Nevertheless, the increase in collagen fibril diameter is correlated with higher Zn levels and even more strongly with aligned collagen. The larger collagen diameter near the metastatic site is in line with the behaviour reported in cell culture which seems to be related to the creation of migration pathways by cancer cells in the surrounding ECM. It paves the way for migration to the circulatory system (intravasation) and, later on to a secondary organ, by increasing the cross-sectional area of interfibrillar pores^54^. The movement of migrating cancer cells to a secondary organ occurs through fibre-like channels created by the highly oriented collagen fibrils perpendicular to the tumour cells (TACS-3), which is supported by a higher correlation between collagen orientation and larger fibril diameter. This collagen remodelling occurs not only in the primary tumour but also during the development of pre-metastatic niches^55^. Such collagen orientation in the tumour microenvironment is induced, among other molecules, by the zinc-dependent endopeptidases MMPs^56^. Therefore, the strongest correlation exhibited in Table 1 between highly oriented collagen and zinc accumulation is in agreement with previous studies^57,58^.

## Conclusions

The advanced X-ray multimodal computed tomography exploited in this work, which encompasses XRF-CT and SAXS-TT, allows, for the first time, obtaining a comprehensive picture of some of the main hallmarks in breast cancer development in a volume-resolved manner. Although significant insights into breast cancer initiation, progression and metastasis have been gained from 2D culture cell or animal models, the findings from this work represent a step forward in shedding light on breast cancer metastasis. In particular, the potential of the advanced multimode X-ray Imaging points in the direction of understanding the collaborative influence of the main key molecules in the sophisticated multifaceted metastatic mechanism when additional breast tumour subtypes are investigated. The strong cross-correlation between zinc accumulation, associated with MMPs, and highly oriented collagen was highlighted. MMPs trigger collagen alignment perpendicular to the tumour cells in the same way as they degrade the ECM, creating a local fibre-like pathway to guide breast cancer cell intravasation. In addition, iron accumulation, mainly associated with angiogenesis and VEGF, provides the necessary blood supply for cell proliferation and metastasis.

As in other types of cancer, the debut of metastasis in breast cancer greatly increases lethality. Less than 1% of the tumour cells undergo this process, but it contributes to more than 90% of cancer-related deaths. By bridging the gap between experimental findings in culture models and animal studies and real tissue samples, this study opens up the field for further investigation of breast cancer metastasis and pre-metastatic niches (PMNs). For example, using advanced X-ray multimodal tomography to generate complementary 3D maps at the local tumour and the secondary organ for distinct breast cancer molecular subtypes. In addition, it can also contribute to the development of more targeted and effective strategies for diagnosing and treating breast cancer metastasis. A comprehensive understanding of the 3D architecture of the tumour microenvironment, in conjunction with collagen orientation and diameter and metal accumulation, may guide the development of novel therapeutic approaches and enable personalised treatment strategies, ultimately improving patient outcomes in breast cancer management.

Convinced of the considerable potential inherent in the presented advanced imaging approach, the subsequent phase entails a thorough exploration of various breast cancer subtypes, including triple-negative and HER2-positive cases. This investigation aims to provide a comprehensive understanding of the metastatic mechanisms in breast cancer. While the expansion of the sample size is constrained by the higher attenuation of Fe-Kα, a significant trace element in biological tissues, optimizing data acquisition and processing can facilitate probing a statistically significant number of samples. Currently, the implementation of the proposed imaging approach is contingent upon state-of-the-art instrumentation and substantial resources, limiting its use to a select few large-scale facilities. However, anticipated advancements in instrumentation, coupled with progress in machine learning, hold the potential to expedite data acquisition and enhance the 3D reconstruction process. These developments may ultimately enhance accessibility, making the innovative imaging technique applicable to a broader array of laboratories and clinical settings.

## Methods

### Case selection and sample preparation

A human breast specimen classified as invasive breast carcinoma of no special type (IBC-NST), based on the World Health Organization (WHO) criteria, was used in this work. The selected case was chosen due to the histological grade III, the presence of lymph node metastases, and the molecular subtype HR−/HER2+. The combination of those features has been shown to exhibit higher content and organization of collagen as peritumoral as intratumoral^59^. In addition, due to the presence of metastatic lymph nodes, there is expected a re-ordering of collagen fibrils and the accumulation of zinc and iron in the neighbourhood of the metastatic foci. The sample is the remaining piece of a surgically extracted specimen for biopsy at the Clinic and Polyclinic for Gynecologic in the Medical University Hamburg-Eppendorf, Hamburg, Germany. Just after excision, a thin slice was cut to obtain prior histopathology information about the samples. An experienced breast pathologist directed the histological examination. The information obtained was taken as a guideline to drive the selection of the region of interest for the X-ray imaging experiments. The remaining portion was immediately frozen at − 80 °C. The excision as the handling of the sample is in accordance with the guidelines of the Declaration of Helsinki and its revisions and approved by the Ethics Commission Hamburg, WF-049/09. The experiment was performed with the remaining part of the tissues used for diagnosis and no personal information from the patient was shared with the researchers, therefore, due to the retrospective nature of the study, the need of informed consent was waived by Ethics Commission Hamburg—Universitätsklinikum Hamburg-Eppendorf.

A few days before the experiment, the region of interest was cut out from the specimen in a cylindrical shape of 1.2 mm diameter and 5 mm height. Afterwards, this sample was submitted to a freeze-drying process to avoid volume variation and minimize radiation-induced damage during the experiment. This process was carried out in the freeze-dryer VaCo-2 from Zirbus GmBh with the condenser at − 80 °C. In the first 72 h, the primary drying was performed by slowly increasing the temperature from − 80 °C to room temperature (22 °C), while the chamber pressure was kept at 2 Pa. In the secondary drying, the temperature and the pressure are maintained constant at room temperature and 2 Pa, respectively, for 24 h. Once the lyophilization process is finished, the vials are immediately filled with nitrogen gas, the sample is sealed in proper flasks and stored at 4 °C. Just before the imaging experiments, the dried sample were brought to room temperature and placed into a Kapton^®^ tube.

### X-ray multimodal computed tomography

**Figure 3:**
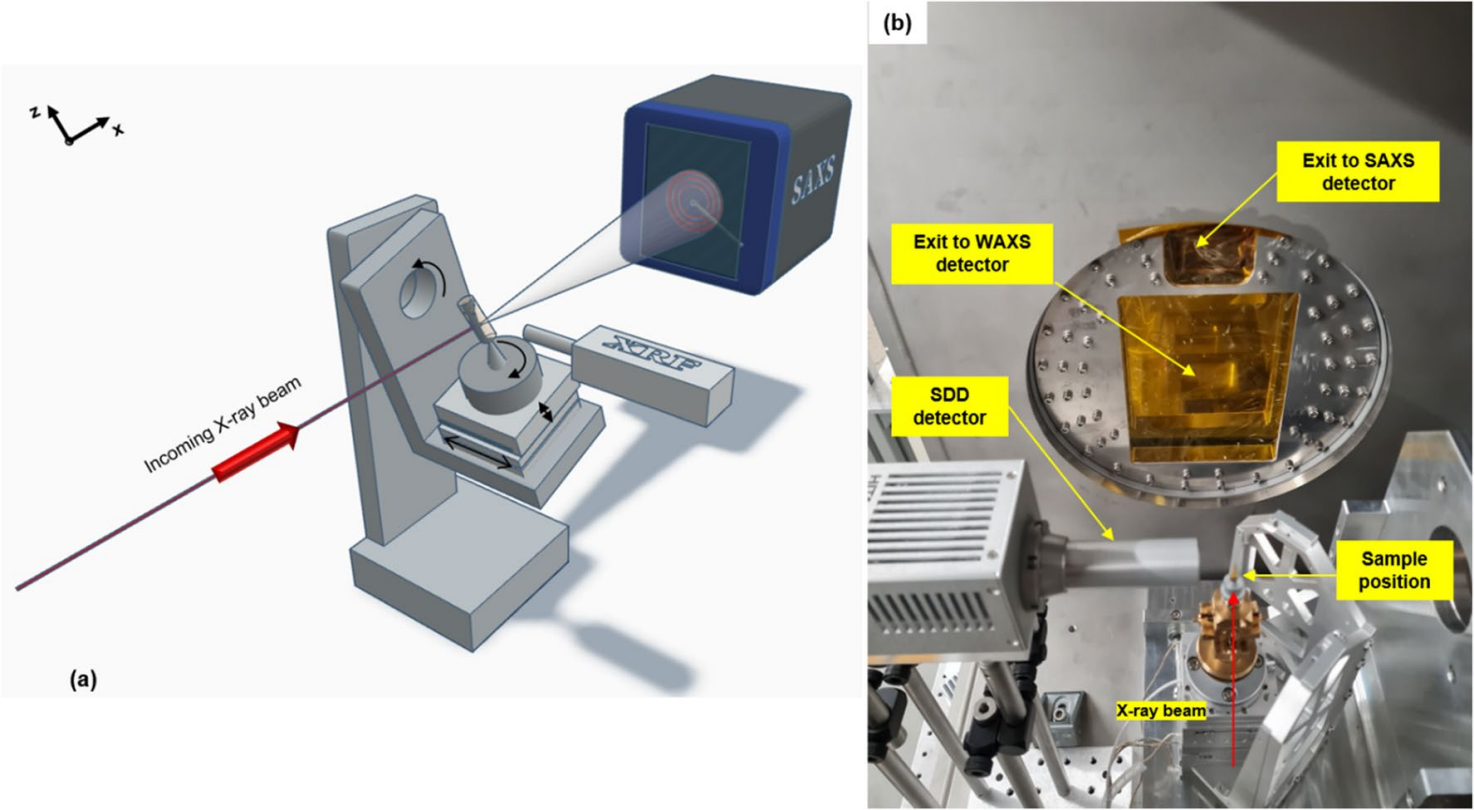
X-ray multimodal computed tomography. (**a**) sketch of the multimodal setup showing the motion system with all the degrees of freedom required for the SAXS Tensor Tomography technique and the coordinate system. The energy dispersive detector for XRF is positioned as close as possible to the sample perpendicularly to the incoming X-ray beam. Finally, the SAXS detector is mounted downstream to the beam at a 5.08 m distance from the sample. A 6.0 mm diameter active beamstop is used to protect the detector of the primary beam as well as to record the transmitted intensity. reconstructed transmission image; (**b**) Photo of the top view of the interior of the chamber filled up with He, presenting the dedicated sample environment for scanning-based tomography at the SAXSMAT beamline at the Deutsches Elektronen-Synchrotron DESY, Hamburg, Germany.

The integrated X-ray fluorescence and small-angle scattering computed tomography allow for mapping the correlation between the accumulation of specific trace elements and the organization of hierarchical structures in a sample three-dimensionally. In this study, the X-ray multimodal setup was assembled at the SAXSMAT beamline, PETRA III storage ring in Hamburg, Germany. The SAXSMAT beamline has a dedicated sample environment for scanning-based X-ray tomography, which can exploit absorption, fluorescence, and small and wide-angle scattering contrast mechanisms simultaneously^60^. In particular, SAXS/WAXS tensor tomography experiments can also be carried out at the beamline. The schematic representation of the multimodal setup utilized in this work, combining X-ray fluorescence tomography and SAXS tensor tomography is represented in Fig. 3a. An X-ray beam with 12.4 keV energy, defined using a Si (111) double crystal monochromator, is focused (25 × 25 µm^2^) on the sample, by a combination of bendable mirror and compound refractive lenses system. The sample, inserted into a Kapton tube, is positioned on the top of a goniometer head mounted in a 4 + 4 degree of freedom motion system. An energy-dispersive silicon drift detector (SDD) from Vortex^®^ with 30 mm^2^ active area is placed 14 mm from the rotation around the z-axis and perpendicular to the incoming X-ray beam to collect the X-ray fluorescence emission from the sample. The motion system and the SDD are inside a chamber filled with He to minimize air background scattering for the SAXS measurements (see Fig. 3b). The small-angle X-ray scattered photons by the sample are recorded with a 2D single photon counting detector Eiger2 9M from Dectris^®^ downstream distant 5.08 m from the sample, in an evacuated flight tube, which allows recording the momentum transfer range 0.05 nm^−1^ < $q$ (= 4πsin(θ/2)/λ) < 2.15 nm^−1^, where θ is the scattering angle and λ the wavelength. An ionization chamber, before the sample, and an active 6.0 mm diameter beamstop, in front of the SAXS detector, were used to monitor the primary and transmitted beam intensities, respectively.

The sample is continuously raster scanned with a virtual spot size of 25 × 25 µm^2^ and 100 ms exposure time per virtual point. The exposure time was calculated to maximize the counts on the SAXS detector within the limit imposed by radiation damage, previously tested in the remaining piece of the same breast sample imaged. A total of 51 SAXS/XRF projections (48 y-steps × 60 z-steps) between 0° and 180° at the upright position (tilt = 0°) were acquired. Additional 245 SAXS projections between 0° and 360° at the tilt angles 9°, 18°, 27°, 36°, and 45° were also recorded, according to the Liebi et al. 2018 approach^61^. In total, 908,925 SAXS patterns and 146,880 XRF spectra were collected. Despite each spot recording an absorbed dose of approximately 1.1 MGy, the structural details essential for precise analysis and interpretation of the scattering data remain intact (refer to the figure in the supplementary section). Notably, no significant alteration is discernible in the scattering profile. This observation is likely attributed to the sample preparation method utilizing freeze-drying, as discussed in the work by Conceição et al.^62^.

### Data processing and image reconstruction

#### SAXS tensor tomography

In the first step, each 2D SAXS pattern was radially integrated in every one of the 16 azimuthal segments between 0° and 360° using the Python package pyFAI^63^. The integration parameters were previously determined through the SAXS pattern of the standard silver behenate. Afterwards, the one-dimensional SAXS profiles were corrected by self-attenuation, effective exposure time per virtual point, normalized by the incoming flux, and the background signal.

The SAXS tensor tomography reconstruction of the collagen fibrils in the breast sample was performed using the Python-based software MUMOTT^64^. MUMOTT relies on band-limited spherical functions to enable the reconstruction of reciprocal space maps of anisotropic nanostructures in a volume-resolved manner^32^. Although all the momentum transfer values were reconstructed, the 5^th^ reflection order of collagen ($q$ ≈ 0.48 nm^−1^) was used to reconstruct the collagen tensor rank-2 map. Since all the q-values were reconstructed the reverse analysis approach, presented in a previous publication^38^ was applied in a region-of-interest of the reconstructed image to retrieve the correspondent SAXS profiles and determine the fibril diameter.

#### XRF computed tomography

The detector channels of the measured XRF spectra were converted to energy using the XRF signal from the calibration standard A-13 of the International Atomic Energy Agency (IAEA) using the software pyMCA^65^. The pyMCA was also used to fit the zinc and iron (Kα and Kβ) emission peaks in every XRF spectrum collected. Later, the area of the fitted peaks at every scanned virtual point was normalized by exposure time and incoming x-ray flux. The transmission map, obtained from the relation between the primary beam registered by the ionization chamber and the transmitted beam recorded by the active beamstop, was used to correct the self-absorption effects. Self-absorption effects refer to the absorption of the incident beam on its transit through the specimen and the absorption of the X-ray fluorescence on its transit to the energy-dispersive detector^66^. The element-specific maps were reconstructed using a standard filtered back-projection (FBP) algorithm (Hamming filter).

### Supplementary Information

Supplementary Figure 1.

## Data Availability

The datasets used and/or analysed during the current study available from the corresponding author on reasonable request.
