# Supplementary material for: Unveiling breast cancer metastasis through an advanced X-ray imaging approach
Source: Sci Rep. 2024 Jan 16;14:1448. doi: 10.1038/s41598-024-51945-4 (PMC10791658; doi:10.1038/s41598-024-51945-4)
Supplement: Supplementary file 1 — Supplementary Figure 1. [file 41598_2024_51945_MOESM1_ESM.pdf]

SUPLEMENTARY

Investigating breast cancer metastasis through collagen remodelling and metal accumulation: an advanced X-ray imaging approach

Andre L.C. Conceição[a*], Volkmar Müller[b], Eike-Christian Burandt[c], Malte Mohme[d], Leonard C. Nielsen[e], Marianne Liebi[e,f,g] and Sylvio Haas[a]

[a] *Deutsches Elektronen-Synchrotron DESY, Notkestr. 85, 22607 Hamburg, Germany*

[b] Department of Gynecology - *University Medical Center Hamburg-Eppendorf, Martinistraße 52, 20246 Hamburg, Germany*

[c] *Institute of Pathology - University Medical Center Hamburg-Eppendorf, Martinistraße 52, 20246 Hamburg, Germany*

[d] *Department of Neurosurgery - University Medical Center Hamburg-Eppendorf, Martinistraße 52, 20246 Hamburg, Germany*

[e] *Department of Physics, Chalmers University of Technology, 41296 Gothenburg, Sweden*

[f] *Photon Science Division, Paul Scherrer Institute, 5232 Villigen PSI, Switzerland*

[g] *Institute of Materials, Ecole Polytechnique Fédérale de Lausanne (EPFL),1015 Lausanne, Switzerland*

* Corresponding Author at: *Deutsches Elektronen-Synchrotron DESY, Notkestr. 85, 22607 Hamburg, Germany.*
Phone: +49-40-8998-5573
E-mail: andre.conceicao@desy.de (A.L.C. Conceição)

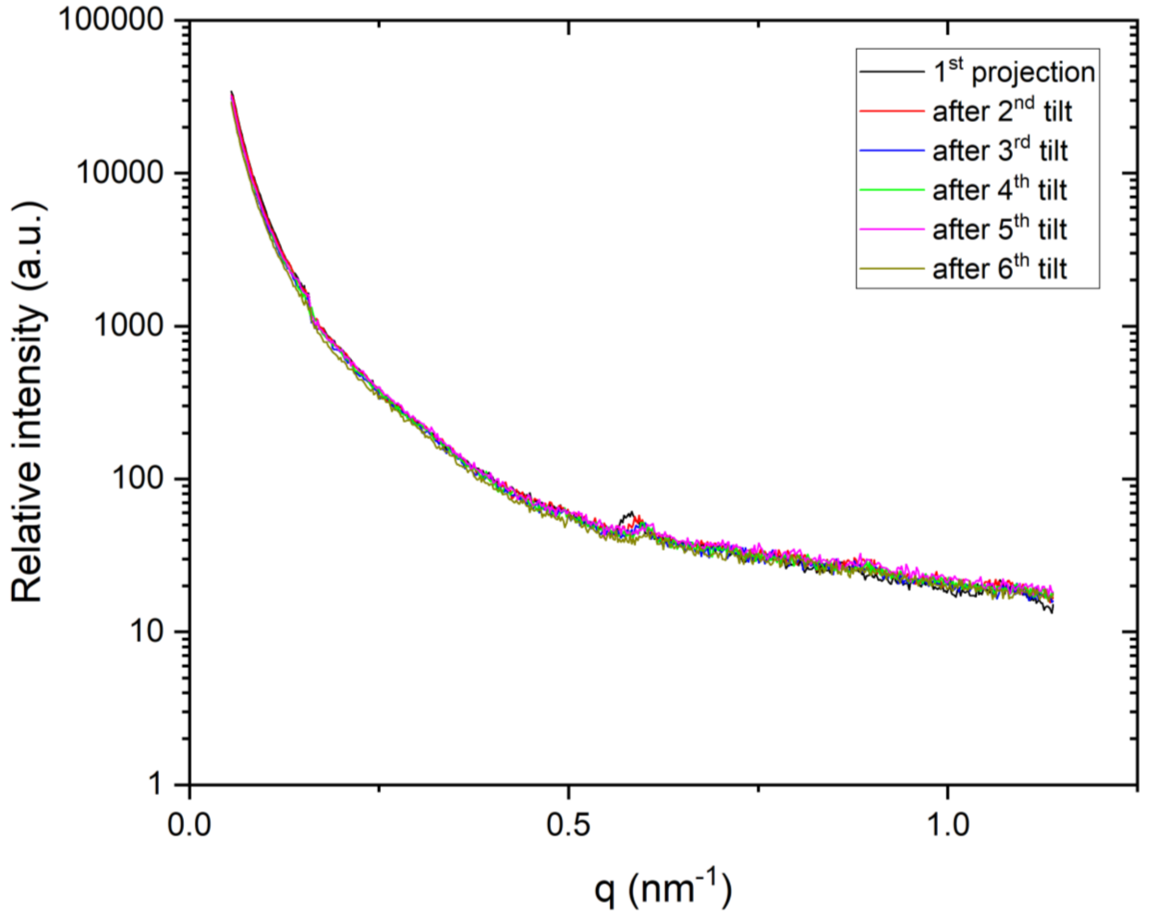

Figure: Scattering profile of the same spot on the sample at the first projection and after each tilt angle acquired for the SAXS-TT.

The SAXS profiles presented in the figure above were obtained at the spatial coordinate at rotation 0° and tilt 0° at the first projection and after each measured tilt. It is clearly, observed no significant alteration in the scattering profile, even after an estimated absorbed dose of 1.1 MGy.
